# Endocrine complications after solid organ transplantation in childhood and adolescents

**DOI:** 10.3389/fendo.2025.1658780

**Published:** 2025-09-11

**Authors:** Ji-Hee Yoon, Dohyung Kim, Soojin Hwang, Ja Hye Kim, Jin-Ho Choi

**Affiliations:** ^1^ Department of Pediatrics, Bundang Jesaeng Hospital, Daejin Medical Center, Seongnam, Gyeonggi-do, Republic of Korea; ^2^ Department of Pediatrics, Asan Medical Center, University of Ulsan College of Medicine, Seoul, Republic of Korea

**Keywords:** solid organ transplantation, short stature, obesity, delayed puberty, dyslipidemia, post-transplant diabetes mellitus

## Abstract

**Objective:**

Acute or chronic metabolic derangement following solid organ transplantation (SOT) often leads to endocrine complications, which have become more common as survival rates post-SOT have improved. This study was performed to investigate long-term endocrine complications after SOT in children and adolescents.

**Methods:**

This study included 259 pediatric patients who underwent SOT, including kidney (n = 43), liver (n = 170), lung (n = 5), heart (n = 37), and multi-organ (n = 4), with a minimum follow-up period of 5 years post-transplant. Clinical and endocrinological data were retrospectively collected, including information on growth, obesity, diabetes, dyslipidemia, thyroid disease, bone health, and pubertal development.

**Results:**

Of 259 patients, 203 (78.4%) developed endocrine complications over a median follow-up period of 10.5 years (range, 5.5–16.8). Short stature was common in kidney (58.1%) and multi-organ recipients (100%), whereas the highest rates of obesity were observed in liver recipients (43.5%). Kidney or liver recipients under 13 years of age showed significant improvements in height-standard deviation scores within 5 years post-SOT. Discontinuation of corticosteroids was associated with a reduced risk of short stature 10 years after liver transplantation. Heart recipients had a high prevalence of post-transplant diabetes mellitus (PTDM, 27%). Other endocrine complications included dyslipidemia (40.2%), hypothyroidism (2.8%), and low bone mineral density (31.3%). Among liver recipients, pretransplant obesity was a significant risk factor for development of post-transplant obesity, PTDM, and dyslipidemia. Additionally, liver transplantation at 0–1 years of age increased the risk of obesity, while transplantation at 6–12 years of age, cyclosporine use, and allograft rejection were associated with an increased risk of dyslipidemia.

**Conclusions:**

This study demonstrates that endocrine and metabolic complications are common in pediatric SOT recipients. Effective surveillance and management of these sequelae are crucial to improve long-term quality of life following SOT.

## 1 Introduction

Solid organ transplantation (SOT), including kidney, liver, lung, and heart, is considered a life-saving treatment for patients with end-stage solid organ failure (1). Advances in donor selection, surgical techniques, and perioperative care, such as immunosuppressive therapy, nutritional support, and infection control, have markedly improved post-transplant outcomes (1–3). Consequently, long-term survival has significantly improved in pediatric SOT, with recent 5-year survival rates reaching 99% for kidney, 85% for heart, and 56.3% for lung transplants (4–7). For liver transplantation, the 20-year survival rate is estimated at 84% (8). Overall, more than 80% of pediatric SOT recipients survive into adolescence and young adulthood (2).

The improved survival rates following SOT in pediatric patients have been accompanied by an increase in endocrine and metabolic complications, which may contribute to cardiovascular disease. These complications are attributed to the reversal of the pretransplant catabolic state, increased body weight, or metabolic effects of immunosuppressants (9). In previous studies on pediatric SOT, up to 30% of kidney transplant recipients experienced obesity or overweight, and 3.5% developed post-transplant diabetes mellitus (PTDM) (10, 11). In liver recipients, obesity and dyslipidemia occurred in 10–17% and 16–50%, respectively (12, 13). Glucose intolerance or PTDM was observed in 3% of liver recipients as early as one month after transplantation (14). In heart recipients, 1.8% experienced PTDM, while up to 52% developed dyslipidemia one year after transplantation (15, 16). Lung recipients with cystic fibrosis exhibited a particularly high risk of PTDM, with a cumulative incidence of 20–30% within 5 years after transplantation (17).

Several studies have reported improvements in height deficit following SOT (18, 19). Patients who underwent kidney transplantation at a young age exhibited catch-up growth during the early post-transplant period (18, 19). Notably, kidney and liver transplantation before the age of 2 years has been associated with the most significant catch-up growth (19–21). Height Z-scores tend to improve gradually after transplantation, generally plateauing around 5 years post-transplantation, with minimal further improvement thereafter (19, 22). A positive effect on height growth has also been observed in kidney and liver recipients following the discontinuation of corticosteroids (22, 23). However, overall growth outcomes remain suboptimal in many pediatric solid organ recipients (24). Inadequate catch-up growth following SOT may result from multiple factors, including poor nutritional status, allograft dysfunction, immunosuppressive therapy, and older age at the time of transplantation (24). Despite these findings, the impact of SOT on long-term growth trajectories remains to be discovered.

Although long-term complications are recognized in pediatric SOT recipients, few studies have comprehensively investigated endocrine complications across multiple organ types in large cohorts with extended follow-up periods. Given the shared pathophysiological mechanisms of endocrine complications–such as exposure to immunosuppressive agents, nutritional alterations, and organ-specific recovery dynamics–an integrated analysis encompassing all major organ types is warranted. This comprehensive approach enables a more thorough understanding of endocrine sequelae, facilitates the identification of risk factors, and supports the development of unified surveillance and intervention strategies. Therefore, this study aimed to evaluate the frequency, clinical characteristics, and risk factors of long-term endocrine complications following SOT in children and adolescents.

## 2 Materials and methods

### 2.1 Subjects

From January 2007 to December 2017, a total of 294 pediatric patients underwent SOT at Asan Medical Center, Seoul, Korea. The inclusion criteria were as follows: 1) patients who underwent kidney, liver, lung, or heart transplantation before the age of 18 years; and 2) patients who were alive and had been followed for a minimum of 5 years post-SOT. Patients whose grafts were removed because of allograft dysfunction were excluded. After excluding 33 patients who died and 2 patients with graft removal, 259 patients were included.

### 2.2 Data collection and endocrine assessment

The following data were retrospectively collected: sex, age at transplantation, underlying disease, anthropometric measurements, and use of immunosuppressive agents such as prednisolone, tacrolimus, cyclosporine, mycophenolate mofetil, and interleukin-2 receptor antibodies (basiliximab and daclizumab). The presence of acute and chronic allograft rejection was pathologically confirmed through tissue biopsy.

The height-standard deviation score (SDS) was assessed annually for the first 5 years and 10 years post-transplantation. Short stature was defined as a height-SDS < -2 according to the age- and sex-matched normative data (25). For children ≥ 2 years of age, obesity was defined as a body mass index (BMI) above the 95th percentile. In children under 2 years of age, obesity was defined as a BMI above the 97.7th percentile (26). Height- and BMI-SDS were calculated using the 2017 Korean National growth charts, based on sex- and age-specific L, M, and S parameters (25).

A diagnosis of hypergonadotropic hypogonadism was established based on elevated luteinizing hormone (LH) and follicle-stimulating hormone (FSH) levels with low testosterone or estradiol levels (27). Delayed puberty was defined as the absence of secondary sexual characteristics after the age of 14 years in males and 13 years in females (28, 29). Functional hypogonadotropic hypogonadism was defined as delayed pubertal maturation attributable to an underlying disease, with spontaneous progression of puberty during the follow-up period and no biochemical or clinical evidence of a permanent reproductive endocrine disorder (30).

PTDM was defined by the presence of any of the following criteria: 1) hemoglobin A1c (HbA1c) levels above 6.5% at least 45 days after SOT to exclude potential confounding factors during the early post-transplant period; 2) fasting serum glucose levels ≥ 126 mg/dL; 3) 2-hour postprandial glucose levels ≥ 200 mg/dL; or 4) random glucose levels ≥ 200 mg/dL with classic symptoms of hyperglycemia such as polydipsia, polyuria, and unexplained weight loss (31). Dyslipidemia was defined as the presence of any of the following: 1) serum total cholesterol ≥ 200 mg/dL; 2) low-density lipoprotein (LDL) cholesterol ≥ 130 mg/dL; triglyceride levels ≥ 130 mg/dL in children aged 10–19 years or ≥ 100 mg/dL in those aged 0–9 years; or 3) high-density lipoprotein (HDL) cholesterol < 40 mg/dL (32).

Primary hypothyroidism was defined as elevated serum thyroid-stimulating hormone (TSH) levels (≥10 μIU/mL) with low or normal free thyroxine levels, whereas subclinical hypothyroidism was defined as serum TSH levels of 4.5–10 μIU/mL with a normal free thyroxine levels (33).

Osteoporosis was defined as the presence of one or more vertebral compression fractures without high-energy trauma, or as the presence of both a clinically significant fracture history and a bone mineral density (BMD) Z-score ≤ –2.0 in the lumbar spine or femur neck according to the age- and sex-matched normative data (34–37). Children with a BMD Z-score ≤ –2.0 without fracture history were categorized as having low BMD (37, 38). BMD of the femoral neck and lumbar spine (L1–L4) was measured using dual-energy X-ray absorptiometry (DXA, Lunar Corp., Madison, WI, USA).

### 2.3 Statistical analysis

Statistical analyses were performed using SPSS for Windows version 21.0 (IBM Corp., Armonk, NY, USA). Continuous variables are presented as mean ± standard deviation (SD) for normally distributed data, and as median and range for skewed distributions. Differences among organ groups were assessed using the Kruskal–Wallis test. Kaplan–Meier curves were used to illustrate the cumulative incidence of endocrine complications, and log-rank tests were applied to compare incidences among organ groups. Changes in height-SDS from baseline to one year post-transplantation were assessed using paired *t*-tests. Annual changes in height-SDS during the first 5 years, as well as changes from baseline to 5 and 10 years after transplantation, were analyzed using repeated measures analysis of variance (ANOVA) with Bonferroni correction for *post hoc* pairwise comparisons. Factors associated with catch-up growth were assessed by multiple linear regression analysis. Logistic regression analysis was used to calculate odds ratios (ORs) with 95% confidence intervals (CIs) for short stature at 5 and 10 years post-transplantation. A multivariate Cox proportional hazards model was employed to estimate hazard ratios (HRs) for endocrine complications after SOT. Thirteen patients underwent the same organ transplantation two or more times due to graft failure; for these cases, the first transplant was defined as the baseline. For multi-organ transplant recipients, the first transplantation was also defined as the baseline. Results were considered statistically significant when *p-*values < 0.05.

## 3 Results

### 3.1 Baseline characteristics of patients who underwent solid organ transplantation

**Figure 1:**
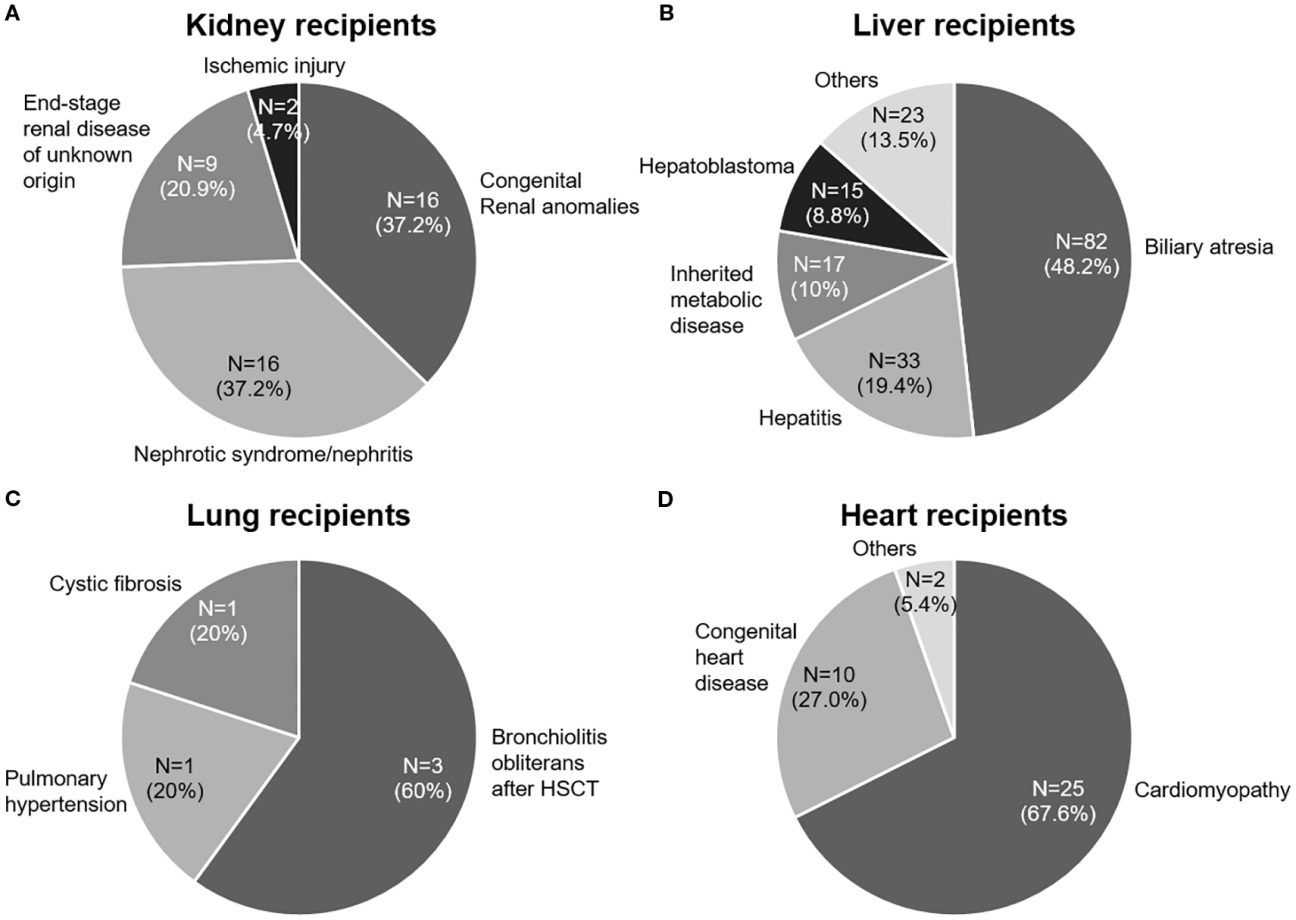
Underlying end-stage organ diseases in pediatric patients who underwent solid organ transplantation (SOT). **(A)** Kidney transplant recipients. **(B)** Liver transplant recipients. **(C)** Lung transplant recipients. **(D)** Heart transplant recipients. HSCT, hematopoietic stem cell transplantation. Inherited metabolic diseases include Wilson’s disease, urea cycle disorders, methylmalonic acidemia, and glycogen storage diseases.

The median age at the time of SOT was 3.9 years (range, 0.3–17.9), with liver recipients being significantly younger than recipients of other organs (*p* < 0.001). The median follow-up duration was 10.5 years (range, 5.5–16.8). The baseline clinical characteristics of the 259 solid organ recipients, categorized by organ type (kidney [n = 43], liver [n = 170], lung [n = 5], heart [n = 37], and multi-organ [n = 4]), are summarized in **Table 1**. The underlying disorders of the patients are shown in **Figure 1**.

**Table 1:** Baseline characteristics of patients who underwent solid organ transplantation (SOT).

Baseline parameters	Kidney (n = 43)	Liver (n = 170)	Lung (n = 5)	Heart (n = 37)	Multi-organ^a^ (n = 4)
Sex					
Male, n (%)	28 (65)	84 (49)	2 (40)	21 (57)	2 (50)
Female, n (%)	15 (35)	86 (51)	3 (60)	16 (43)	2 (50)
Median age at SOT (months, range)	158 (20–213)	18 (3–167)	184 (140–198)	133 (5–215)	113.5 (5–189)
Median follow-up duration (months, range)	129 (77–202)	134 (66–201)	80 (73–141)	121 (75–198)	126 (104–172)
Anthropometrics, Z-scores					
Height, mean (SDS)	-2.0 (1.75)	-0.6 (1.74)	-1.4 (2.15)	-1.0 (1.60)	-3.3 (1.96)
Weight, mean (SDS)	-2.0 (1.78)	-0.3 (1.45)	-1.5 (1.99)	-1.8 (1.60)	-3.0 (2.20)
BMI^b^, mean (SDS)	-1.2 (1.79)	0.2 (1.44)	-1.1 (2.30)	-1.5 (1.23)	-0.4 (1.40)
Short stature at baseline, n (%)	19 (44)	30 (18)	2 (40)	8 (22)	3 (75)
Obesity at baseline, n (%)	0 (0)	19 (11.0)	0 (0)	0 (0)	0 (0)
DM at baseline, n (%)	1 (2.3)	0 (0)	2 (40)	0 (0)	0 (0)
Thyroid disease at baseline, n (%)	2 (4.7)	2 (1.2)	1 (20)	2 (5.4)	0 (0)
Baseline immunosuppressive drugs					
Corticosteroid, n (%)	43 (100)	170 (100)	5 (100)	37 (100)	4 (100)
Tacrolimus, n (%)	37 (86)	170 (100)	5 (100)	29 (78)	4 (100)
Cyclosporine, n (%)	6 (14)	0 (0)	0 (0)	8 (22)	0 (0)
Mycophenolate mofetil, n (%)	36 (84)	106 (62)	5 (100)	37 (100)	4 (100)
Basiliximab/Daclizumab, n (%)	43 (100)	139 (82)	5 (100)	37 (100)	4 (100)
Maintenance immunosuppressive drugs					
Tacrolimus based, n (%)	33 (77)	162 (96)	4 (80)	23 (62)	4 (100)
Cyclosporine based, n (%)	7 (16)	7 (4)	1 (20)	5 (14)	0 (0)
mTOR inhibitors, n (%)	3 (7)	1 (1)	0 (0)	6 (16)	0 (0)
Discontinuation of steroid, n (%)	24 (56)	119 (70)	0 (0)	34 (92)	2 (50)
Allograft rejection, n (%)	24 (56)	97 (57)	0 (0)	19 (62)	1 (25)

SDS, standard deviation score; SOT, solid organ transplantation; BMI, body mass index; DM, diabetes mellitus; mTOR. mammalian target of rapamycin

a Patients in the multi-organ transplant group included those who underwent liver and kidney transplantation (n = 2), heart and kidney transplantation (n = 1), and combining lung, heart, and kidney transplantation (n = 1).

b BMI was calculated for patients aged 2 years and older.

All patients were initially treated with methylprednisolone after transplantation and were subsequently switched to either prednisolone or deflazacort (Calcort^®^, Handok Inc., Seoul, Korea). The initial immunosuppressive regimen was tacrolimus-based in 245 patients (94.6%) and cyclosporine-based in 14 patients (5.4%). Corticosteroid therapy was discontinued in 179 patients (69.1%) at a mean of 27.7 ± 32.4 months after SOT, with the highest discontinuation rate observed in the heart transplantation group (n = 34, 92%). Biopsy-confirmed acute or chronic allograft rejection occurred in 141 recipients (54.4%).

### 3.2 Endocrine complications after SOT

**Figure 2:**
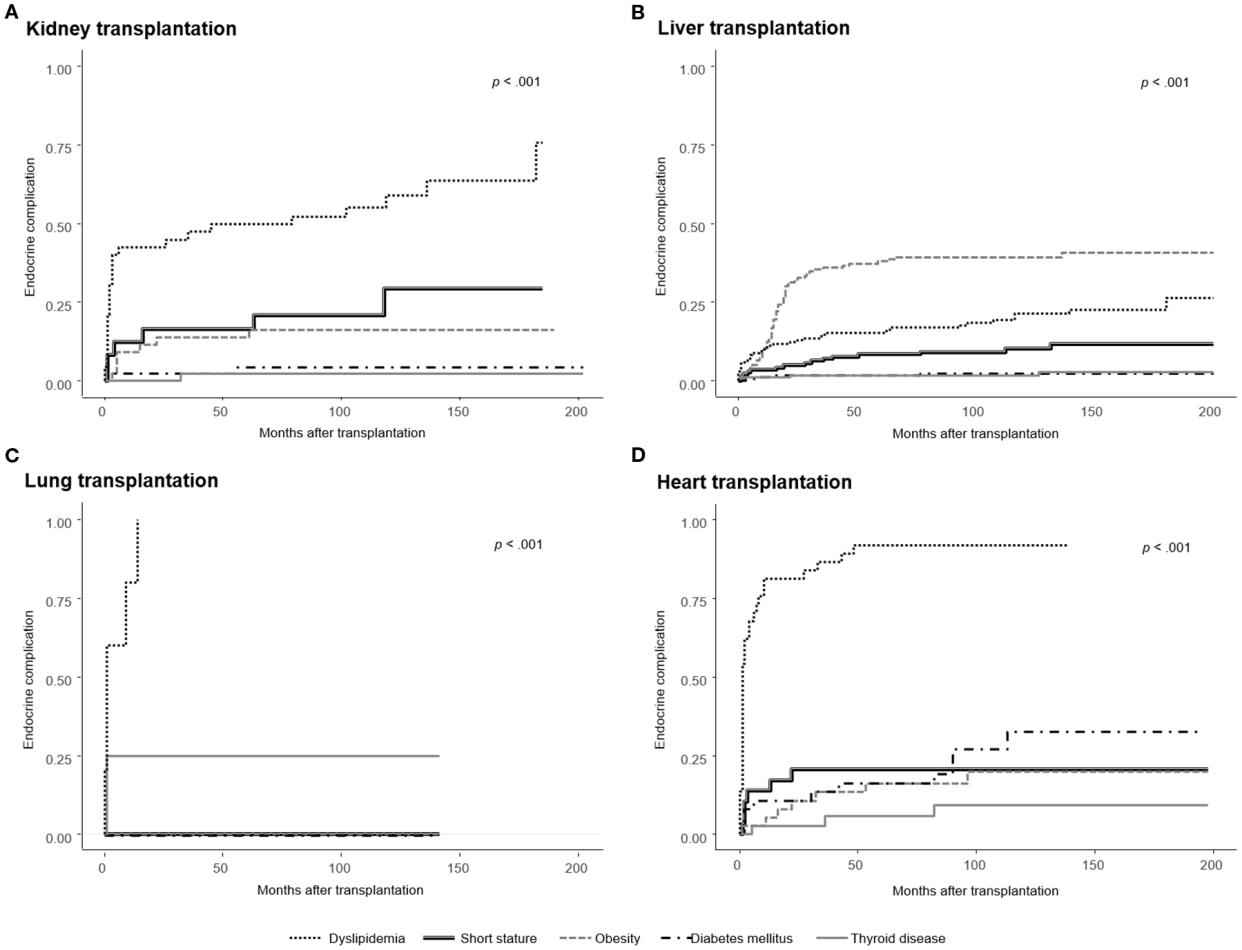
Cumulative incidence of newly developed endocrine complications after solid organ transplantation (SOT), excluding patients with pre-existing conditions. Kaplan–Meier curves are stratified by organ type: **(A)** Kidney transplantation, **(B)** Liver transplantation, **(C)** Lung transplantation, and **(D)** Heart transplantation. The incidence of endocrine complications differed significantly by organ type (log-rank *p* < 0.001), with the highest obesity rates observed in liver recipients, post-transplant diabetes mellitus (PTDM) in heart recipients, and dyslipidemia in both heart and lung recipients. Short stature was most frequent among multi-organ recipients overall; however, among the organ types shown in this figure, kidney recipients had the highest incidence.

Endocrine complications–including short stature, obesity, PTDM, thyroid disorders, dyslipidemia, low BMD, or delayed puberty–were observed in 203 of 259 patients (78.4%), with frequencies varying by the type of transplanted organ: kidney (n = 33, 76.7%), liver (n = 126, 74.1%), lung (n = 5, 100%), heart (n = 35, 94.6%), and multi-organ (n = 4, 100%) (**Table 2**). The cumulative incidences of newly developed endocrine complications after SOT, excluding patients with pre-existing conditions, are illustrated in **Figure 2**. The overall prevalence of endocrine complications differed significantly according to organ type (log-rank *p* < 0.001).

**Table 2:** Endocrine complications following SOT.

Endocrine complications	Kidney (n = 43)	Liver (n = 170)	Lung (n = 5)	Heart (n = 37)	Multi-organ (n = 4)
Overall, n (%)	33 (77)	126 (74)	5 (100)	35 (95)	4 (100)
Short stature, n (%)	25 (58)	43 (25)	2 (40)	13 (35)	4 (100)
Male, n (%)	17 (68)	24 (56)	1 (50)	8 (62)	2 (50)
Female, n (%)	8 (32)	19 (44)	1 (50)	5 (38)	2 (50)
Obesity, n (%)	7 (16)	74 (44)	0 (0)	7 (19)	1 (25)
Male, n (%)	5 (71)	37 (50)	0 (0)	4 (57)	0 (0)
Female, n (%)	2 (29)	37 (50)	0 (0)	3 (43)	1 (100)
BMI Z-score, mean (SDS)	1.7 (0.05)	1.9 (0.44)	ND	1.9 (0.54)	1.72
DM^a^, n (%)	2 (5)	4 (2)	0 (0)	10 (27)	1 (25)
Drug-induced DM, n (%)	2 (5)	3 (2)	0 (0)	9 (24)	1 (25)
Type 2 DM, n (%)	0 (0)	1 (0.6)	0 (0)	1 (2.7)	0 (0)
Time since SOT (month), mean (SDS)	29.5 (36.06)	25.5 (34.58)	ND	41.5 (38.46)	7
Thyroid disease^a^, n (%)	1 (2)	4 (2)	1 (25)	3 (9)	1 (25)
Primary hypothyroidism, n (%)	0 (0)	2 (1)	0 (0)	2 (6)	1 (25)
Central hypothyroidism, n (%)	1 (2)	0 (0)	1 (25)	0 (0)	0 (0)
Hyperthyroidism, n (%)	0 (0)	2 (1)	0 (0)	1 (3)	0 (0)
Time after SOT (month), mean (SDS)	32	37.5 (60.52)	1	41.0 (38.74)	7
Dyslipidemia, n (%)	26 (60)	36 (21)	5 (100)	34 (92)	3 (75)
Hypertriglyceridemia, n (%)	19 (44)	33 (19)	4 (80)	33 (89)	3 (75)
Hypercholesterolemia, n (%)	15 (35)	10 (6)	3 (60)	11 (30)	0 (0)
Total cholesterol (mg/dL), mean (SDS)	226.2 (67.81)	177.6 (99.72)	262.0 (61.21)	201.2 (66.20)	113.3 (38.08)
Triglyceride (mg/dL), mean (SDS)	189.3 (75.69)	202.1 (91.40)	428.0 (505.07)	211.7 (112.12)	277.0 (35.68)
HDL-cholesterol (mg/dL), mean (SDS)	59.7 (28.60)	52.5 (26.16)	44.8 (15.61)	57.3 (16.54)	ND
LDL-cholesterol (mg/dL), mean (SDS)	131.7 (57.54)	111.9 (32.41)	154.2 (33.15)	119.7 (52.69)	ND
Time since SOT (month), mean (SDS)	29.9 (53.26)	35.6 (48.22)	5.0 (6.20)	5.2 (10.44)	1.0 (1.73)
Low BMD, n (%)	2 (15)	32 (29)	5 (100)	1 (50)	2 (67)
L-spine BMD Z-score, mean (SDS)	-3.2 (1.09)	-1.9 (1.09)	-3.1 (1.37)	-1.4	-3.35 (0.07)
Femur BMD Z-score, mean (SDS)	-3.9 (0.22)	-2.3 (1.11)	-4.1 (2.97)	-4.5	-1.7 (6.58)
Time since SOT (month), mean (SDS)	26.5 (37.48)	112.1 (44.03)	26.8 (28.60)	56.0	38.5 (50.20)
Delayed puberty, n (%)	2 (5)	0 (0)	1 (20)	0 (0)	1 (25)
Male, n (%)	2 (100)	0 (0)	1 (100)	0 (0)	1 (100)
Female, n (%)	0 (0)	0 (0)	0 (0)	0 (0)	0 (0)
Hypergonadotropic hypogonadism, n (%)	1 (2)	0 (0)	1 (20)	0 (0)	0 (0)
Hypogonadotropic hypogonadism, n (%)	1 (2)	0 (0)	0 (0)	0 (0)	0 (0)

BMD, Bone mineral density; BMI, body mass index; DM, diabetes mellitus; HDL, high-density lipoprotein; LDL, low-density lipoprotein; SDS, standard deviation score; SOT, solid organ transplantation; ND, No data.

**^a^** Recipients who were diagnosed with DM and thyroid disease before SOT were excluded.

#### 3.2.1 Short stature

At baseline, the mean height-SDS of multi-organ transplant recipients was significantly lower than that of recipients of other organs (*p* < 0.001; **Table 1**). During the follow-up period, short stature was observed in 87 patients (33.6%). It was most prevalent among multi-organ recipients (n = 4, 100%), followed by kidney (n = 25, 58.1%), lung (n = 2, 40.0%), heart (n = 13, 35.1%), and liver recipients (n = 43, 25.3%). Among 10 patients with underlying syndromic disorders, including Alagille syndrome, Turner syndrome, and Noonan syndrome, 9 patients (90%) exhibited with short stature, either at baseline or developing after transplantation.

**Figure 3:**
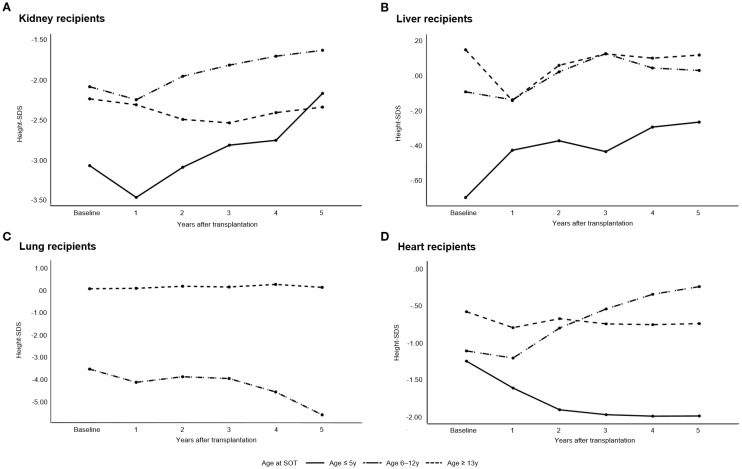
Annual changes in height standard deviation scores (height-SDS) during the first 5 years following pediatric solid organ transplantation (SOT). **(A)** Kidney transplant recipients, **(B)** Liver transplant recipients, **(C)** Lung transplant recipients, and **(D)** Heart transplant recipients. Kidney recipients showed a significant decline in height-SDS during the first year post-transplantation; however, those transplanted before the age of 13 years demonstrated significant catch-up growth by year 5 (*p* = 0.003). Liver recipients transplanted before the age of 13 years showed significant improvement at 5 years (*p* < 0.001). Heart recipients aged 6–12 years demonstrated significant increases in height-SDS in the first 5 years (*p* = 0.027).

**Figure 3** illustrates the annual changes in height-SDS during the first 5 years following pediatric SOT. Among kidney transplant recipients, the mean height-SDS significantly decreased during the first year post-transplantation (from -2.0 ± 1.8 to -2.3 ± 1.8; *p* = 0.043). However, by 5 years post-transplantation, patients who underwent kidney transplantation before the age of 13 years showed a significant improvement in height-SDS (*p* = 0.003). In multiple linear regression analysis, younger age at kidney transplantation was significantly associated with greater height gain at 5 years post-transplantation (*p* = 0.016). No further significant change in height-SDS was observed between 5 and 10 years post-transplantation (*p* = 0.105, repeated measures ANOVA).

In patients who underwent liver transplantation before the age of 13 years, height-SDS improved significantly at both 5 and 10 years post-transplantation (*p* < 0.001 and *p* = 0.003, respectively). Among the 104 patients who were followed up at least 10 years, the mean height-SDS at 10 years was 0.04 ± 1.5 (median age: 11.5 years; range: 10–27.8). Logistic regression analysis demonstrated that discontinuation of corticosteroid therapy was independently associated with a reduced risk of short stature at 10 years post-transplantation (OR = 0.116, *p* = 0.039; **Table 3**). Other organ groups were excluded from this analysis due to insufficient sample sizes for meaningful statistical interpretation.

**Table 3:** Logistic regression analysis of short stature at 5th and 10th year post-transplantation.

Parameters	5 years after SOT		10 years after SOT	
	OR (95% CI)	*p-*value	OR (95% CI)	*p-*value
Kidney transplantation^a^				
Short stature at baseline	10.736 (2.217–51.988)	.003*	2.436 (0.149–39.781)	.532
Liver transplantation^b^				
Short stature at baseline	8.044 (2.443–26.490)	<.001*	3.042 (0.376–24.614)	.297
Discontinuation of corticosteroid	0.352 (0.093–1.336)	.125	0.116 (0.015–0.901)	.039*

CI, confidence interval; OR, odds ratio; SOT, solid organ transplantation.

a Adjusted for sex, age at transplant (months), baseline and maintenance immunosuppressive drugs (tacrolimus, cyclosporine, and mTOR inhibitors), discontinuation of steroids, allograft rejection, need for re-dialysis, and obesity at baseline.

b Adjusted for sex, age at transplant (months), baseline and maintenance immunosuppressive drugs (tacrolimus, cyclosporine, and mTOR inhibitors), allograft rejection, need for retransplantation, and obesity at baseline.

* Statistically significant at *p* < 0.05.

Heart transplant recipients aged 6–12 years at the time of transplantation showed significant improvement in height-SDS during the first 5 years post-transplantation (*p* = 0.027); however, no significant change was observed between 5 and 10 years post-transplantation (*p* = 0.280).

Recombinant human growth hormone (rhGH) therapy was administered to 23 patients prior to transplantation, predominantly among kidney recipients (n = 20). Following transplantation, rhGH treatment was continued in 5 patients (four kidney recipients and one heart recipient), and newly initiated in 2 patients (one heart recipient and one lung recipient). Although height-SDS tended to improve from −4.0 ± 2.4 to −3.3 ± 2.9, this change was not statistically significant (*p* = 0.138). One heart recipient who initiated rhGH therapy 57 months after transplantation developed post-transplant lymphoproliferative disorder 9 months later, resulting in discontinuation of rhGH treatment.

#### 3.2.2 Obesity, diabetes mellitus, and dyslipidemia

Obesity was observed in 89 patients (34.4%) after SOT. On average, obesity developed 21.8 ± 22.0 months post-transplantation (range, 1–137 months). The cumulative incidence of obesity was significantly higher among liver recipients compared to recipients of other organs (61/151, 40.4%, log-rank *p* = 0.008), with most cases occurring within the first 5 years after SOT. **Table 4** presents the risk factors for post-transplant metabolic complications in liver recipients. Kidney, heart, lung, and multi-organ recipients were excluded from this analysis due to insufficient sample sizes for reliable statistical evaluation. Among liver recipients, obesity at the time of transplantation was a significant risk factor for post-transplant obesity (HR = 2.81, *p* = 0.001) (**Table 4**). Additionally, patients who underwent liver transplantation at 0–1 years of age had a significantly higher risk of developing obesity compared to those transplanted at 6–12 year of age (HR = 2.24, *p* = 0.035).

**Table 4:** Multivariate Cox proportional hazard analysis of endocrine complications following SOT.

Parameters	Obesity		Post-transplant diabetes mellitus		Dyslipidemia	
	HR (95% CI)	*p-*value	HR (95% CI)	*p-*value	HR (95% CI)	*p-*value
Liver transplantation						
Obesity at baseline	2.81 (1.52–5.21)	0.001*	10.26 (1.40-75.38)	0.022*	5.60 (2.65–11.83)	<.001*
At age 0–1 years	2.24 (1.06–4.75)	0.035*	NA		1.00^c^	Ref
At age 2–5 years	1.50 (0.61–3.71)	0.376	NA		2.18 (0.89–5.33)	0.088
At age 6–12 years	1.00^a^	Ref	NA		2.42 (1.03–5.72)	0.044*
At age 13–18 years	1.00 (0.30–3.39)	0.994	NA		1.49 (0.46–4.77)	0.505
Maintenance treatment with tacrolimus	1.00^b^	Ref	NA		1.00^d^	Ref
Maintenance treatment with cyclosporine	1.57 (0.56–4.39)	0.389	NA		3.98 (1.19–13.31)	0.025*
Allograft rejection	1.22 (0.75–2.00)	0.427	NA		3.03 (1.30–7.06)	0.010*
Discontinuation of corticosteroids	0.81 (0.49–1.35)	0.418	0.30 (0.04–2.22)	0.239	0.80 (0.38–1.67)	0.552

CI, confidence interval; HR, hazard ratio; SOT, solid organ transplantation; NA, not assessed; Ref, reference category.

a Reference category for age was 6–12 years.

b Reference category for maintenance treatment was tacrolimus.

c Reference category for age was 0–1 years.

d Reference category for maintenance treatment was tacrolimus.

*Statistically significant at *p* < 0.05.

PTDM developed in 17 patients (6.6%), with the highest proportion observed among heart recipients (27.0%). In patients with PTDM, the mean random serum glucose and HbA1c levels were 208.6 ± 104.6 mg/dL and 8.0 ± 1.6%, respectively. The mean time to PTDM onset was 38.5 ± 35.9 months following SOT. Two cases of type 2 diabetes mellitus were diagnosed in obese patients: one with a BMI-SDS of 4.0 following liver transplantation and another with a BMI-SDS 3.2 following heart transplantation. Among patients with PTDM, 11 were receiving insulin therapy and 2 were receiving metformin at the time of diagnosis. One patient with type 2 diabetes mellitus was managed with both metformin and insulin treatment. Among liver recipients, obesity prior to transplantation was strongly associated with the development of PTDM (HR = 10.26, *p* = 0.022) (**Table 4**). No cases of type 1 diabetes mellitus were observed.

Dyslipidemia developed in 104 patients (40.2%), with particularly high frequencies observed among lung and heart recipients–100% (n = 5) and 92% (n = 34), respectively. Among liver recipients, pre-transplant obesity and allograft rejection were identified as significant risk factors for dyslipidemia (HR = 5.60, *p* < 0.001; HR = 3.03, *p* = 0.010, respectively) (**Table 4**). Patients who underwent liver transplantation aged 6–12 years had a significantly higher risk of developing dyslipidemia compared to those transplanted at 0–1 year of age (HR = 2.42, *p* = 0.044). Additionally, a cyclosporine-based immunosuppressive regimen was associated with an increased risk of dyslipidemia compared to tacrolimus (HR = 3.98, *p* = 0.025).

#### 3.2.3 Hypothyroidism, low BMD, and delayed puberty

Central hypothyroidism was diagnosed in 2 patients with acute lymphoblastic leukemia who had previously undergone total body irradiation before hematopoietic stem cell transplantation and subsequently developed bronchiolitis obliterans requiring lung transplantation. Both patients were treated with levothyroxine. Additionally, one kidney recipient and one liver recipient exhibited non-thyroidal illness. Subclinical hypothyroidism developed in 21 patients post-transplantation. In most cases (n = 18, 86%), including 2 patients with Turner syndrome and Noonan syndrome, thyroid function normalized spontaneously during follow-up; however, 3 patients (14%) with TSH levels ≥ 10 μIU/mL required levothyroxine replacement therapy.

DXA was performed consecutively in 134 recipients who were referred to the Department of Pediatric Endocrinology for evaluation of bone mass. Low BMD was observed in 42 of these 134 recipients (31.3%), including kidney (n = 2), liver (n = 32), lung (n = 5), heart (n = 1), and multi-organ recipients (n = 2). However, none of the patients met the criteria for pediatric osteoporosis.

Delayed puberty was observed in 4 male recipients. One kidney recipient was diagnosed with hypergonadotropic hypogonadism, attributed to underlying Klinefelter syndrome. Additionally, functional hypogonadotropic hypogonadism was diagnosed in 3 patients: one kidney recipient, one lung recipient, and one multi-organ recipient.

A summary table linking major endocrine complications to organ type and modifiable risk factors is provided in **Supplementary Table 1**.

## 4 Discussion

In this study, 78.4% of solid organ recipients experienced at least one endocrine complication, although the frequency varied by organ type. Lung and multi-organ recipients exhibited the highest frequency (100%). Among kidney recipients, short stature was the most common complication (58.1%), whereas obesity was relatively common among liver recipients (43.5%). Among lung recipients, dyslipidemia (100%) and low BMD (100%) were major complications, whereas PTDM was frequent in heart recipients (27%).

Growth outcomes varied by organ type and age at transplantation. Among kidney recipients, undergoing transplantation before puberty was associated with a significant increase in height-SDS during the first 5 years post-transplantation, and younger age at kidney transplantation correlated with greater height gain, consistent with a previous study (19). In the present study, liver recipients—who were significantly younger at transplantation (median age at SOT: 18 months)— exhibited sustained catch-up growth at both 5 and 10 years post-transplantation, in contrast to some previous reports of insufficient growth recovery (39, 40). Conversely, heart recipients transplanted before the age of 6 years did not demonstrate significant catch-up growth. Across organ groups, recipients in this younger age range had markedly lower baseline height-SDS, reflecting their vulnerability to prolonged disease burden. In heart recipients, where disease severity is high and chronic post-transplant nutritional challenges are common, even early transplantation may not fully reverse pre-existing growth impairment (41), underscoring the need for organ-specific strategies to optimize growth outcomes.

In the present study, rhGH therapy after SOT was infrequently used, and no statistically significant improvement in height-SDS was observed, likely due to the small number of treated patients. Previous studies have demonstrated that rhGH can improve growth velocity in pediatric kidney and liver transplant recipients and is not associated with an increased risk of adverse events (42, 43). However, our findings also underscore potential safety concerns, as one heart transplant recipient who initiated rhGH 57 months post-transplant developed post-transplant lymphoproliferative disorder 9 months after the initiation of rhGH therapy. This highlights the importance of careful patient selection, risk-benefit assessment, and close monitoring when considering rhGH therapy in pediatric SOT recipients.

Glucocorticoids affect the growth plate by suppressing chondrocyte proliferation, reducing bone formation, and altering endochondral ossification (7, 44). They also promote calcium loss through the kidneys and gastrointestinal tract, and interfere with the growth hormone (GH)/insulin-like growth factor (IGF) axis by down-regulating GH receptors and inhibiting IGF-1 synthesis (45, 46). The beneficial effect of corticosteroid discontinuation on growth was evident in liver recipients, in whom cessation of corticosteroids was associated with a significantly reduced risk of short stature at 10 years post-transplantation, consistent with previous studies (22, 47).

Obesity was most commonly observed within the first 5 years following transplantation, with baseline overweight status identified as a significant predictor of post-transplant obesity, consistent with previous studies (48, 49). The development of obesity can be attributed to the resolution of the catabolic state induced by organ failure, increased appetite following disease recovery, and corticosteroid use after SOT (9, 50). While a recent study reported the highest incidence of obesity in pediatric kidney recipients, our study found that liver recipients had the highest prevalence of obesity (48). To optimize pretransplant status, aggressive nutritional rehabilitation is often recommended for children with severe malnutrition associated with end-stage liver disease (51). In the present study, 11% of liver transplant recipients were already obese at the time of transplantation. Notably, 55% of liver recipients underwent transplantation at 0–1 years of age, and approximately 50% of these infants developed obesity during the follow-up period. In the multivariate Cox analysis, this age group demonstrated a significantly increased risk of developing obesity compared to those transplanted at 6–12 years of age. BMI percentiles increased markedly after SOT, and rapid weight gain, particularly during infancy, was associated with an increased risk of childhood obesity (49, 52). These findings suggest careful monitoring and individualized nutritional strategies may be warranted to avoid overfeeding in this vulnerable population.

Immunosuppressive drugs can cause dyslipidemia within the first year following SOT (53, 54). Cyclosporine is a well-established risk factor for dyslipidemia, as it inhibits hepatic synthesis of the LDL receptors, interferes with LDL cholesterol binding, and decreases bile acid synthesis, thereby impairing cholesterol clearance. An additive effect may also occur when cyclosporine is used in combination with corticosteroids (55, 56). In contrast, tacrolimus has a favorable effect on serum lipid profiles (57). In our study, dyslipidemia was the most common endocrine complication following SOT (40.2%), and cyclosporine-based immunosuppression was associated with an increased risk of dyslipidemia in liver recipients. This vulnerability may be further exacerbated in children who underwent liver transplantation between the ages of 6 and 12 years–a period corresponding to the pubertal transition, which is characterized by physiological insulin resistance and impaired insulin-mediated suppression of free fatty acid oxidation (58). These metabolic alterations, when combined with the dyslipidemic effects of immunosuppressive therapy, may synergistically increase the risk of post-transplant dyslipidemia.

PTDM occurred in 6.6% of patients, with a mean onset of approximately 3 years after transplantation. The highest incidence was observed among heart recipients. PTDM is associated with premature cardiovascular morbidity and mortality in adult SOT recipients (59–61). The increase in visceral adiposity after SOT, leading to chronic low-grade inflammation and insulin resistance, is strongly associated with the development of PTDM (62). Corticosteroids decrease peripheral insulin sensitivity, impair insulin synthesis/secretion, and increase hepatic gluconeogenesis (63). Notably, calcineurin inhibitors such as tacrolimus can cause a dose-dependent reduction in insulin synthesis and sensitivity, as well as pancreatic β-cell toxicity (63). A previous study reported a progressive increase in the cumulative incidence of PTDM over time (64); therefore, regular screening for PTDM is recommended following SOT.

Endocrine complications after pediatric SOT including obesity, metabolic syndrome, dyslipidemia, and PTDM, are closely linked to long-term cardiovascular risk. Theses conditions have been shown to accelerate cardiovascular morbidity and mortality in both pediatric and adult transplant populations (65, 66). A recent pediatric study also demonstrated that cardiometabolic risk factors such as obesity, hypertension, and dyslipidemia are prevalent among pediatric kidney transplant recipients and are strongly associated with an increased prevalence of left ventricular hypertrophy (67). In liver transplant survivors, post-transplant metabolic complications and the chronic adverse effects of immunosuppressive therapy are recognized contributors to atherosclerotic cardiovascular disease (68). Collectively, these observations highlight the importance of early recognition and management of endocrine complications as a strategy to reduce cardiovascular risk in this population.

This study has several limitations. Its retrospective design resulted in some missing or inconsistently assessed data, such as anthropometric measurements, blood pressure, Tanner stage, and BMD. In particular, the absence of sufficient blood pressure data precluded a comprehensive evaluation of cardiovascular risk and metabolic complications. Certain transplant- and donor-related factors, as well as pre-transplant kidney replacement therapy details, were also unavailable, limiting a comprehensive analysis of growth determinants. BMI and BMD-SDS values were calculated using chronological rather than height age in patients with a height-SDS < −2, which may have slightly overestimated the prevalence of obesity and low BMD; however, the number of such cases was small. The number of SOT recipients and the duration of follow-up varied. Exclusion of patients who died or underwent graft removal due to allograft dysfunction may have led to an underestimation of the frequency of endocrine complications. As a single-center study, the predominance of liver transplantations and the use of specific immunosuppressive regimens, such as interleukin-2 receptor antagonists instead of anti-thymocyte globulin, may limit generalizability. Additionally, cumulative corticosteroid doses were unavailable, and subgroup analyses of organ-specific differences were limited by small sample sizes.

Despite these limitations, this study has several strengths. It represents one of the largest single-center cohorts to examine a comprehensive range of endocrine and metabolic complications in pediatric SOT recipients with long-term follow-up. By stratifying outcomes by organ type and incorporating growth trajectory analyses, this study provides valuable insights into endocrine sequelae following SOT and highlights modifiable risk factors that may inform future management strategies.

## 5 Conclusions

This study comprehensively characterized long-term endocrine complications in a large, single-center cohort of 259 pediatric SOT recipients. Approximately two-thirds of the cohort experienced at least one endocrine complication. The prevalence of endocrine complications varied by the type of transplanted organ. Notably, prepubertal recipients of kidney and liver transplants exhibited meaningful catch-up growth during long-term follow-up. These findings underscore the need for pediatric endocrinologists to recognize the clinical manifestations and long-term endocrine sequelae associated with SOT. A better understanding of the incidence and risk factors for long-term endocrine complications across all organ groups will facilitate the identification of high-risk populations and support the development of life-long surveillance and targeted prevention strategies.

## Data Availability

The raw data supporting the conclusions of this article will be made available by the authors, without undue reservation.
