# Supplementary material for: Endocrine complications after solid organ transplantation in childhood and adolescents
Source: Front Endocrinol (Lausanne). 2025 Sep 11;16:1658780. doi: 10.3389/fendo.2025.1658780 (PMC12460080; doi:10.3389/fendo.2025.1658780)
Supplement: Supplementary file 1 [file Table1.docx]

**Supplementary Table 1** Summary of major endocrine complications to organ type and modifiable risk factors

| **Transplanted organ type** | **Major endocrine complication** | **Key modifiable risk factors** |
| --- | --- | --- |
| Kidney | Short stature | Baseline short stature; younger age at transplant associated with better growth |
| Liver | Obesity | Pre-transplant obesity associated with obesity, PTDM, and dyslipidemia; younger age at transplant (0–1 yr) associated with higher obesity risk  Age 6–12 yr at transplant, allograft rejection, and cyclosporine (*vs.* tacrolimus) use associated with higher dyslipidemia risk  Earlier steroid discontinuation was beneficial for height |
| Lung | Dyslipidemia, limited catch-up growth |  |
| Heart | PTDM | Pre-transplant obesity associated with PTDM |
| Multi-organ | Short stature (highest overall) |  |
